# Supplementary material for: Tocilizumab demonstrates superiority in decreasing C-reactive protein levels in hospitalized COVID-19 patients, compared to standard care treatment alone
Source: Microbiol Spectr. 2024 Apr 30;12(6):e02498-23. doi: 10.1128/spectrum.02498-23 (PMC11237561; doi:10.1128/spectrum.02498-23)
Supplement: Supplemental material — Fig. S1; Table S1 and S2. [file spectrum.02498-23-s0001.pdf]

Supplemental Material

Figure S1: Decrease over time of CRP is more pronounced after the use of Tocilizumab.

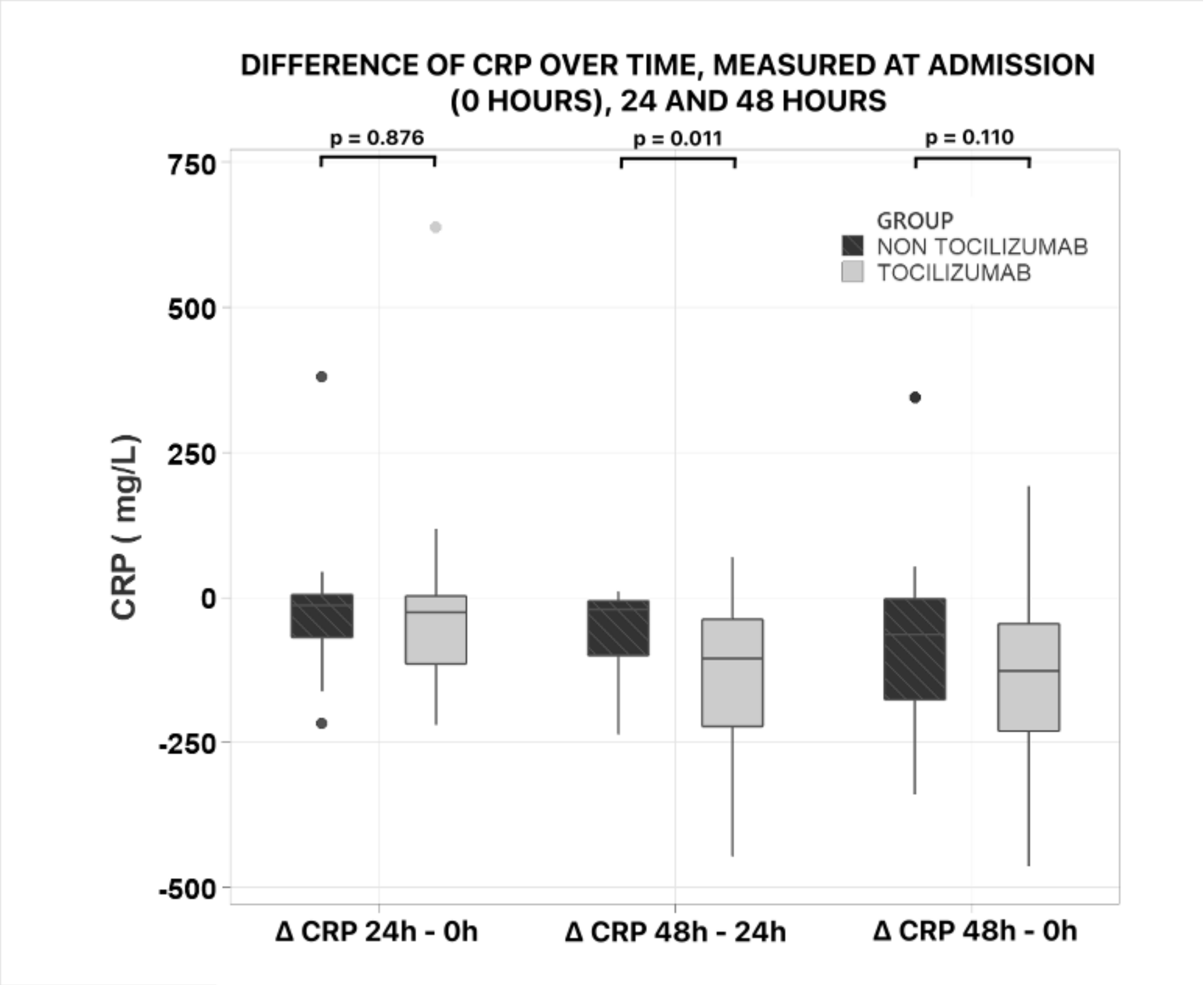

Data are presented as the difference of CRP over time, measured at admission (0 h), 24 and 48 hours, in the Non- Tocilizumab (n = 14) and Tocilizumab group (n = 23). The difference in CRP between two measures is expressed as Δ 24-0h, Δ 48-24h and Δ 48-0h. A Mann-Whitney's test showed a statistical difference when comparing the Δ 48- 24h between both groups, evidencing steeper CRP decrease after the use of Tocilizumab (p = 0.011). The Δ 24-0h and Δ 48-0h were not significant between groups (p = 0.876 and p = 0.110 respectively).

Table S1. Laboratory parameters by group

	All n = 141	Tocilizumab group n = 100	Non-Tocilizumab group n = 41	*p - value*
**Lymphocytes %**	8-18 (14)	7-17 (12.5)	13-26 (16)	<0.001
**Lymphopenia <19%**	109 (77.3%)	84 (84%)	25 (60.9%)	0.003
**NLR**	4-11 (6)	5-61 (7)	3-6 (5)	<0.001
**Neutrophils %**	73-88 (79)	76-88 (81)	63-81 (76)	<0.001
**Ferritin, ng/mL**	277-931 (606)	451-1035 (772)	184-562 (274)	<0.001
**C-Reactive Protein (CPR), mg/L, at admission**	62-293 (200)	85- 297 (219)	27-244 (58.5)	0.015
**CPR >75 mg/L**	93 (65.9%)	76 (76%)	17 (41.1%)	<0.001
**CPR Severity**				<0.001
**Mild (0-14 mg/L)**	8 (5.6%)	4 (4%)	4 (9.7%)	
**Moderate (15-74 mg/L)**	38 (26.9%)	17 (17%)	21 (51.2%)	
**Severe (>75 mg/L)**	92 (65.2%)	76 (76%)	16 (39%)	
**CPR mg/L, IQR (median) after 24 hours admission**	48-254 (123.4)	54.4-254.5 (144.4)	30.7-239.8 (80.2)	0.268
**CPR mg/L, IQR (median) after 48 hours admission**	24.2-98.6 (44.7)	31.2-99.3 (54.1)	18.9-64.6 (21.6)	0.754

Data are expressed as interquartile range IQR (median) or as a number (percentage), unless otherwise noted. Abbreviations: NLR: Neutrophil Lymphocyte Ratio; CRP: C-Reactive Protein.

Table S2. Clinical and demographic characteristics at admission by group

	All<br>n = 141	Tocilizumab group<br>n = 100	Non-Tocilizumab group<br>n = 41	p - value
**Age in years**	47-65 (54)	46-60.5 (52.5)	50-69 (58)	0.270
**Ethnicity:**				0.221
**Hispanic %**	119 (84.3%)	82 (82%)	37 (90.2%)	
**Mennonites %**	22(15.6%)	18 (18%)	4 (9.7%)	
**Comorbidities %**				
**Hypertension**	48 (34%)	35 (35%)	14 (34.1%)	0.923
**T2DM**	39 (27.6%)	28 (28%)	11 (26.8%)	0.888
**Smoking**	32 (22.6%)	21 (21%)	11 (26.8%)	0.453
**Sex:**				
**Masculine %**	94 (66.6%)	69 (69%)	25 (60.9%)	0.359
**Feminine %**	47 (33.3%)	31 (31%)	16 (39%)	
**BMI, Kg/m2**	27-35 (31)	28-35 (31)	26-33 (30)	0.038
**SpO_2 on admission**	83-90 (88)	81-89 (87)	87-92 (90)	0.072
**SpO_2 <92%**	117 (82.9%)	90 (90%)	27 (65.8%)	0.001
**Heart Rate (HR), bpm**	80-104 (90)	82-108 (91)	78-100 (87)	0.137
**HR >90 bpm**	71 (50.3%)	52 (52%)	19 (46.3%)	0.615
**Respiratory Rate (RR), rpm**	20-27 (24)	21-28 (24)	19-24 (21)	0.005
**RR >20 rpm**	102 (72.3%)	77 (77%)	25 (61%)	0.053
**Steroid use**	136 (96.4%)	98 (98%)	38 (92.6%)	0.147
**Dexamethasone**	93 (65.9%)	64 (64%)	30 (73.1%)	0.570
**Metilprednisolona**	64 (45.3%)	55 (55%)	13 (29.2%)	0.035
**Length of hospital stay (HS Length)**	5-11 (7)	6-11 (8)	4-7 (5)	0.124
**Days since symptoms onset until hospitalization**	6-25 (9)	7-10.5 (9)	5-10(7.5)	0.429
**Outcomes**				
**Improvement %**	129 (91.4%)	92 (92%)	37 (90.2%)	0.745
**Death %**	12 (8.5%)	8 (8%)	4 (9.7%)	
**CT score (INER)**	8-16 (11)	9-16 (12)	5-13 (8)	0.002

Data are expressed as interquartile range IQR (median) or as a number (percentage), unless otherwise

noted. Abbreviations: T2DM: Type 2 Diabetes Mellitus; bpm; beats per minute; BMI: Body-Mass Index; IQR: Interquartile Range; SpO2: transdermic oxygen saturation; INER: National Institute of Respiratory Diseases from Spanish Instituto Nacional de Enfermedades Respiratorias.
